# Positive Solutions of Advanced Differential Systems

**DOI:** 10.1155/2013/613832

**Published:** 2013-08-31

**Authors:** Josef Diblík, Mária Kúdelčíková

**Affiliations:** ^1^Department of Mathematics and Descriptive Geometry, Faculty of Civil Engineering, Brno University of Technology, Veveří 331/95, 602 00 Brno, Czech Republic; ^2^Department of Mathematics, Faculty of Electrical Engineering and Communication, Brno University of Technology, Technická 3058/10, 616 00 Brno, Czech Republic; ^3^Department of Mathematics, University of Žilina, Univerzitná 8215/1, 010 26 Žilina, Slovakia

## Abstract

We study asymptotic behavior of solutions of general advanced differential systems $\dot{y}(t)=F(t,y_{t})$, where *F* : Ω → ℝ^*n*^ is a continuous quasi-bounded functional which satisfies a local Lipschitz condition with respect to the second argument and Ω is a subset in ℝ × *C*_*r*_^*n*^, *C*_*r*_^*n*^ : = *C*([0, *r*], ℝ^*n*^), *y*_*t*_∈*C*_*r*_^*n*^, and *y*_*t*_(*θ*) = *y*(*t* + *θ*), *θ* ∈ [0, *r*]. A monotone iterative method is proposed to prove the existence of a solution defined for *t* → ∞ with the graph coordinates lying between graph coordinates of two (lower and upper) auxiliary vector functions. This result is applied to scalar advanced linear differential equations. Criteria of existence of positive solutions are given and their asymptotic behavior is discussed.

## 1. Introduction

Let *C*([*a*, *b*], ℝ^*n*^), where *a*, *b* ∈ ℝ, *a* < *b*, be the Banach space of the continuous mappings from the interval [*a*, *b*] to ℝ^*n*^ equipped with the supremum norm

(1) $$\begin{array}{c} {||\psi ||}_{C}=\underset{\theta \in \left[a,b\right]}{\sup}||\psi \left(\theta \right)||,\;\psi \in C\left(\left[a,b\right],{\mathbb{R}}^{n}\right), \end{array}$$

where ||·|| is the maximum norm in ℝ^*n*^. In the case of *a* = 0 and *b* = *r* > 0, we will denote this space as *C*_*r*_^*n*^; that is,

(2) Crn:=C([0,r],ℝn).

If *σ* ∈ ℝ, *A* ≥ 0, and *y* ∈ *C*([*σ*, *σ* + *r* + *A*], ℝ^*n*^), then, for each *t* ∈ [*σ*, *σ* + *A*], we define *y*_*t*_ ∈ *C*_*r*_^*n*^ by *y*_*t*_(*θ*) = *y*(*t* + *θ*), *θ* ∈ [0, *r*].

Let us consider a system of functional differential equations of advanced type:

(3) $$\begin{array}{c} \dot{y}\left(t\right)=F\left(t,y_{t}\right), \end{array}$$

where *F* : *Ω* → ℝ^*n*^ is a continuous quasi-bounded functional which satisfies a local Lipschitz condition with respect to the second argument and *Ω* is a subset in ℝ × *C*_*r*_^*n*^. We recall that the functional *F* is quasi-bounded if *F* is bounded on every set of the form [*t*_1_, *t*_2_] × *C*_*rL*_^*n*^ ⊂ *Ω*, where *t*_1_ < *t*_2_, *C*_*rL*_^*n*^ : = *C*([0, *r*], *L*), and *L* is a closed bounded subset of ℝ^*n*^ (cf. [1, page  305]).

A function *y* is said to be a *solution of* (3) *on *[*σ*, *σ* + *A*) with *A* > 0 if *y* ∈ *C*([*σ*, *σ* + *r* + *A*], ℝ), (*t*, *y*_*t*_) ∈ *Ω* for *t* ∈ [*σ*, *σ* + *A*), and *y*(*t*) satisfies (3) for *t* ∈ [*σ*, *σ* + *A*).

The paper is organized as follows. In Section 2 necessary iterative technique is formulated. In Section 3 it is applied to general nonlinear advanced differential system. By monotone iterative method, we will prove a general criterion on existence of bounded solutions of the nonlinear system (3) or, more exactly, we give necessary conditions for the existence of a solution defined for *t* → *∞* with the graph coordinates lying between graph coordinates of two (lower and upper) auxiliary vector functions. If the lower function can be taken with positive coordinates, then the statement of theorem concerns positive solutions.

Section 4 is devoted to scalar linear cases. Nonlinear result proved in Section 3 is applied to the scalar advanced linear differential equation

(4) $$\begin{array}{c} \dot{y}\left(t\right)=\left(c+d\left(t\right)\right)y\left(t+r\right), \end{array}$$

where *c*, *r* ∈ ℝ^+^ = (0, *∞*) are constants and *d*(*t*) is a locally Lipschitz continuous function satisfying |*d*(*t*)|<*ɛ* < *c* for *t* ∈ [*t*_0_, *∞*). We will assume (*c* + *ɛ*) < 1/(*re*) as well. The case when there exist positive solutions is studied. With the aid of two auxiliary equations, constructed using lower and upper estimates of the right-hand side of (4), and under the supposition of the existence of two real (positive) different roots of corresponding transcendental equations, the existence of a positive solution of (4) is proved. Simultaneously its asymptotic behavior is derived. Next, a linear advanced equation, more general than (4),

(5) $$\begin{array}{c} \dot{y}\left(t\right)=\gamma \left(t\right)y\left(t+r\right), \end{array}$$

where *γ* : [*t*_0_, *∞*) → ℝ^+^ is bounded, locally Lipschitz continuous function and *r* is a positive constant, is considered and a criterion of existence of positive solutions is given.

The fact of existence of positive solutions of an advanced differential equation can be documented, for example, by the equation

(6) $$\begin{array}{c} \dot{y}\left(t\right)=\frac{1}{\tau e}y\left(t+\tau \right) \end{array}$$

which admits a pair of positive and asymptotically different for *t* → *∞* solutions:

(7) $$\begin{array}{c} y_{1}\left(t\right)=t\exp\left(\frac{t}{\tau }\right),\;\;y_{2}\left(t\right)=\exp\left(\frac{t}{\tau }\right). \end{array}$$

In the literature one can find some results on existence of positive solutions of advanced equations. For example, in accordance with [2, page  31] and [3, page  21], the first-order advanced type differential equation

(8) $$\begin{array}{c} \dot{y}\left(t\right)=p\left(t\right)y\left(t+\tau \left(t\right)\right), \end{array}$$

where *p*, *τ* ∈ *C*([*t*_0_, *∞*), ℝ^+^), has a positive solution in the case when *τ*(*t*) ≡ *τ* > 0 if and only if there exists a continuous function *λ*(*t*) such that

(9) $$\begin{array}{c} \int_{t}^{t+\tau }p\left(u\right)du\leq \Lambda^{-1}\left(\Lambda \left(t\right)+\ln\lambda \left(t\right)\right)-t, \end{array}$$

where Λ(*t*) = ∫_*t*_0__^*t*^ *λ*(*s*)*ds* and Λ^−1^ is corresponding inverse function.

As a consequence of our result for nonlinear advanced equations we get a sufficient and necessary criterion of positivity different from the above-mentioned criterion (9).

Concluding remarks and open problems are formulated in Section 5.

## 2. Preliminaries

In this section we introduce some definitions and theorems which will be used later.

**Definition 1 (see [4, page 276])**

Let *𝕃* be a Banach space and let *𝕂*⊆*𝕃*. Then *𝕂* is called an order cone if and only if

- *𝕂*  is closed, nonempty, and *𝕂* ≠ {0};
- *α*, *β* ∈ *R*, *α*, *β* ≥ 0, *x*, *y* ∈ *K*⇒*αx* + *βy* ∈ *𝕂*;
- *x* ∈ *𝕂* and −*x* ∈ *𝕂*⇒*x* = 0.

On this basis, we define for *x*, *y* ∈ *𝕂*

(10) x≤y iff  y−x∈𝕂,x<y iff  x≤y  and  x≠y,x≪y iff  y−x∈int⁡(𝕂),x≰y iff  x≤y  is  false,[x,y]:={z∈𝕃:x≤z≤y}  (order  interval).

**Definition 2 (see [4, page 277])**

Let *𝕃*, *𝕐* be Banach spaces and let *𝕂*⊆*𝕃* be an order cone.

(1) The order cone *𝕂* is called *normal* if and only if there is a number *c* > 0 such that, for all *x*, *y* ∈ *𝕃*,
  (11) $$\begin{array}{c} \text{If}\;0\leq x\leq y\;\text{then}\;||x||\leq c||y||. \end{array}$$
(2) The operator *T* : *D*(*T*)⊆*𝕃* → *𝕐* is called *monotone increasing* if and only if it is true for all *x*, *y* ∈ *D*(*T*) that
  (12) $$\begin{array}{c} x<y\;\text{implies}\;Tx\leq Ty, \end{array}$$

where *D*(*T*) denotes domain of definition of the operator *T*. The operator is called *strictly* or *strongly* monotone increasing if and only if the symbol “≤” is replaced by “<” or “≪,” respectively. If we replace “≤” by “≥,” then *T* is monotone decreasing. Similarly, we have operators which are strictly or strongly monotone decreasing.

### 2.1. Supersolutions, Subsolutions, and Iterative Methods

We consider the operator equation

(13) $$\begin{array}{c} x=Tx \end{array}$$

together with two iterative methods

(14) $$\begin{array}{c} u_{n+1}=Tu_{n},\;\;v_{n+1}=Tv_{n}. \end{array}$$

**Definition (see [4, page 282])**

The point *x* is called a supersolution, a strict supersolution, or a strong supersolution of (13) if and only if *x* ≥ *Tx*, *x* > *Tx*, or *x* ≫ *Tx*, respectively. The prefix “super” is replaced by “sub” when the respective inequalities are reversed.

**Definition 4 (see [4, page 54])**

Let *𝕃*, *𝕄* be Banach spaces, and *T* : *D*(*T*)⊆*𝕃* → *𝕄* an operator. *T* is called compact if and only if

- *T* is continuous;
- *T* maps bounded sets into relatively compact sets.

Let us recall that a set *𝔸* is *bounded* if and only if there is a number *R* > 0 such that ||*x*|| ≤ *R* for all *x* ∈ *𝔸* and is *relatively compact* (resp., *compact*) if and only if every sequence in *𝔸* contains a convergent subsequence (resp., the limit of which also belongs to *𝔸*).

The following theorem is a part of Theorem 7.A in [4, page  283].

**Theorem 5**

Suppose that *T* : [*u*_0_, *v*_0_]⊆*𝕃* → *𝕃* is a compact monotone increasing operator on a real Banach space *𝕃* with normal order cone *𝕂*. If *u*_0_ is a subsolution of (13) and if *v*_0_ is a supersolution of (13) with *u*_0_ ≤ *v*_0_, then the iterative sequence {*v*_*n*_} in (14) converges to a fixed point of *T*, namely, to the greatest fixed point *v* of *T* in [*u*_0_, *v*_0_], and {*u*_*n*_} converges to the smallest fixed point *u* of *T* in [*u*_0_, *v*_0_]. Furthermore, one has the error estimates

(15) $$\begin{array}{c} u_{n}\leq u\leq v\leq v_{n},\;\forall n=0,1,\ldots . \end{array}$$

## 3. Nonlinear Case

In this part the advanced system of differential equations (3) is considered. Using the method of monotone sequences we prove that under certain additional assumptions there exists a solution of (3) satisfying half-infinity interval inequalities formulated in Theorem 6 (inequalities (26)).

By ℝ_≥0_^*n*^  (ℝ_>0_^*n*^) we denote the set of all component-wise nonnegative (positive) vectors *v* in ℝ^*n*^; that is, *v* = (*v* ^1^,…, *v* ^*n*^) with *v* ^*i*^ ≥ 0 (*v* ^*i*^ > 0) for *i* = 1,…, *n*. For *u*, *v* ∈ ℝ^*n*^, we say that *u* ≤ *v* if *v* − *u* ∈ ℝ_≥0_^*n*^, *u* ≪ *v* if *v* − *u* ∈ ℝ_>0_^*n*^, *u* < *v* if *u* ≤ *v*, and *u* ≠ *v*.

For a fixed t* ∈ ℝ we put *Ω* : = (t*, *∞*) × *C*_*r*_^*n*^. In the following, let *t*_0_ ∈ ℝ and *t*_0_ > t*.

Now for a given *k* ∈ ℝ_>0_^*n*^, we consider two systems of the integrofunctional inequalities

(16) $$\begin{array}{c} \lambda_{1}\left(t\right)\leq {\left(I\left(k,\lambda_{1}\right)\left(t\right)\right)}^{-1}F\left(t,I{\left(k,\lambda_{1}\right)}_{t}\right),\\ \lambda_{2}\left(t\right)\geq {\left(I\left(k,\lambda_{2}\right)\left(t\right)\right)}^{-1}F\left(t,I{\left(k,\lambda_{2}\right)}_{t}\right) \end{array}$$

on [*t*_0_, *∞*), *t*_0_ ∈ ℝ^*n*^ for *λ*_1_, *λ*_2_ ∈ *C*([*t*_0_, *∞*), ℝ^*n*^), *λ*_1_(*t*) < *λ*_2_(*t*), where

(17) $$\begin{array}{c} I:{\mathbb{R}}_{>0}^{n}\times C\left(\left[t_{0},\infty \right),{\mathbb{R}}^{n}\right)\to C\left(\left[t_{0},\infty \right),{\mathbb{R}}^{n}\right) \end{array}$$

is defined by

(18) $$\begin{array}{c} I^{i}\left(k,\lambda \right)\left(t\right):=k^{i}\exp\left(\int_{t_{0}}^{t}\lambda^{i}\left(s\right)ds\right), \end{array}$$

where *i* = 1,…, *n*, *t* ∈ [*t*_0_, *∞*), *λ* ∈ *C*([*t*_0_, *∞*), ℝ^*n*^).

The next theorem provides conditions under which it is possible to construct (with the aid of auxiliary given functions *λ*_1_, *λ*_2_ satisfying (16)) monotone sequences of functions converging to a solution of the operator equation

(19) $$\begin{array}{c} \lambda \left(t\right)=\left(T\lambda \right)\left(t\right) \end{array}$$

on [*t*_0_, *∞*) with

(20) $$\begin{array}{c} \left(T\lambda \right)\left(t\right):={\left(I\left(k,\lambda \right)\left(t\right)\right)}^{-1}F\left(t,I{\left(k,\lambda \right)}_{t}\right). \end{array}$$

It is easy to verify that the system (3) is related to operator equation (19) through the substitution

(21) $$\begin{array}{c} y\left(t\right)=I\left(k,\lambda \right)\left(t\right). \end{array}$$

A function *λ* is said to be a solution of operator equation (19) on [*σ*, *σ* + *A*) with *A* > 0 if *λ* ∈ *C*([*σ*, *σ* + *r* + *A*), ℝ^*n*^), (*t*, *λ*_*t*_) ∈ *Ω* for *t* ∈ [*σ*, *σ* + *A*), and *λ*(*t*) satisfies (19) for *t* ∈ [*σ*, *σ* + *A*).

**Theorem 6**

Let one assumes the following.

(i) For any *M* ≥ 0, *ϑ* ≥ *t*_0_ there are *K*_1_, *K*_2_ such that
  (22) $$\begin{array}{c} ||\left(T\lambda \right)\left(t\right)||\leq K_{1}, \end{array}$$
  (23) $$\begin{array}{c} ||\left(T\lambda \right)\left(t\right)-\left(T\lambda \right)\left(t^{\prime }\right)||\leq K_{2}\left|t-t^{\prime }\right| \end{array}$$
  for any *t*, *t*′ ∈ [*t*_0_, *ϑ*] and any function *λ* ∈ *C*([*t*_0_, *ϑ* + *r*], ℝ^*n*^) with ||*λ*||_*C*_ ≤ *M*, where the norm is defined by (1) with *a* = *t*_0_ and *b* = *ϑ* + *r*.
(ii) There is a *k* ∈ ℝ_>0_^*n*^ and functions *λ*_*j*_ ∈ *C*([*t*_0_, *∞*), ℝ^*n*^), *j* = 1,2 satisfying *λ*_1_(*t*) ≤ *λ*_2_(*t*) on [*t*_0_, *∞*) and inequalities (16) on [*t*_0_, *∞*), that is, the inequalities
  (24) λ1(t)≤(Tλ1)(t),λ2(t)≥(Tλ2)(t).
(iii) For any Λ_*j*_ ∈ *C*([*t*_0_, *s* + *r*), ℝ^*n*^), *j* = 1,2 with Λ_1_(*t*) ≤ Λ_2_(*t*) for *t* ∈ [*t*_0_, *s* + *r*), *s* ≥ *t*_0_, one has
  (25) $$\begin{array}{c} \left(T\Lambda_{1}\right)\left(t\right)\leq \left(T\Lambda_{2}\right)\left(t\right),\;t\in \left[t_{0},s\right). \end{array}$$

Then there exists a solution *y* = *y*(*t*) of the system (3) on [*t*_0_, *∞*) satisfying

(26) $$\begin{array}{c} I\left(k,\lambda_{1}\right)\left(t\right)\leq y\left(t\right)\leq I\left(k,\lambda_{2}\right)\left(t\right) \end{array}$$

for *t* ∈ [*t*_0_, *∞*).

**Proof**

We prove that (19) has a continuous solution *λ* : [*t*_0_, *∞*) → ℝ^*n*^ satisfying *λ*_1_(*t*) ≤ *λ*(*t*) ≤ *λ*_2_(*t*) for *t* ≥ *t*_0_. For every fixed *ϑ* ∈ (*t*_0_ + *r*, *∞*), we introduce the Banach space

(27) $$\begin{array}{c} {𝕃}_{\vartheta }:=C\left(\left[t_{0},\vartheta \right],\;\;{\mathbb{R}}^{n}\right) \end{array}$$

of the continuous functions taking [*t*_0_, *ϑ*] into ℝ^*n*^ equipped with the maximum norm and the normal cone

(28) $$\begin{array}{c} {𝕂}_{\vartheta }:=C\left(\left[t_{0},\vartheta \right],{\mathbb{R}}_{\geq 0}^{n}\right) \end{array}$$

of the continuous functions taking [*t*_0_, *ϑ*] into ℝ_≥0_^*n*^. By the cone *𝕂*_*ϑ*_, a partial ordering ≤ in *𝕃*_*ϑ*_ is given. For *λ*, *μ* ∈ *𝕃*_*ϑ*_ we say that *λ* ≤ *μ* if and only if *μ* − *λ* ∈ *𝕂*_*ϑ*_. We introduce the operator *T*_*ϑ*_ : *𝕃*_*ϑ*_ → *𝕃*_*ϑ*_ defined by

(29) (Tϑλ)(t)={(Tλ)(t)for  t∈[t0,ϑ−r],(Tλ)(ϑ−r)for  t∈[ϑ−r,ϑ].

Let *λ*, *μ* ∈ *𝕃*_*ϑ*_ with *λ* ≤ *μ*. Condition (iii) implies that *T*_*ϑ*_ *λ* ≤ *T*_*ϑ*_ *μ* if *λ* ≤ *μ*. Let

(30) μϑ,j(t):={λj(t)for  t∈[t0,ϑ−r],λj(ϑ−r)for  t∈[ϑ−r,ϑ],j=1,2.

Condition (ii) implies that

(31) μϑ,1(t)≤(Tϑμϑ,1)(t)≤(Tϑ2μϑ,1)(t)≤⋯≤(Tϑ2μϑ,2)(t)≤(Tϑμϑ,2)(t)≤μϑ,2(t),t∈[t0,ϑ].

In order to show the convergence of the sequences

(32) $$\begin{array}{c} {\left(T_{\vartheta }^{m}\mu_{\vartheta ,1}\right)}_{m=0}^{\infty },\;\;{\left(T_{\vartheta }^{m}\mu_{\vartheta ,2}\right)}_{m=0}^{\infty } \end{array}$$

(we set *T*_*ϑ*_^0^ *μ*_*ϑ*,*i*_ = *μ*_*ϑ*,*i*_ and *T*_*ϑ*_^1^ *μ*_*ϑ*,*i*_ = *Tμ*_*ϑ*,*i*_, *i* = 1,2) to the fixed points of operator *T*_*ϑ*_, we need to prove that *T*_*ϑ*_ is continuous and compact. The first property is obvious (due to continuity of *F* and *I*). Let us prove compactness. To this end, let *ℒ* be a bounded subset of *𝕃*_*ϑ*_. We have to show that *T*_*ϑ*_ *ℒ* is a relatively compact subset of *𝕃*_*ϑ*_. Due to the theorem of Arzelà and Ascoli, it suffices to show that *T*_*ϑ*_ *ℒ* is bounded and equicontinuous. That *T*_*ϑ*_ *ℒ* is bounded follows from condition (22), and the equicontinuity of *T*_*ϑ*_ *ℒ* is assumed by condition (23).

Now we are in a position to apply Theorem 5 (about the monotone iterative method) in order to show the existence of the fixed points ${\bar{\mu }}_{\vartheta ,1}$ and ${\bar{\mu }}_{\vartheta ,2}$ of *T*_*ϑ*_ with

(33) $$\begin{array}{c} {\bar{\mu }}_{\vartheta ,1}=\underset{m\to \infty }{\lim}T_{\vartheta }^{m}\mu_{\vartheta ,1},\;\;{\bar{\mu }}_{\vartheta ,2}=\underset{m\to \infty }{\lim}T_{\vartheta }^{m}\mu_{\vartheta ,2},\\ \mu_{\vartheta ,1}\left(t\right)\leq {\bar{\mu }}_{\vartheta ,1}\left(t\right)\leq {\bar{\mu }}_{\vartheta ,2}\left(t\right)\leq \mu_{\vartheta ,2}\left(t\right),\;t\in \left[t_{0},\vartheta \right]. \end{array}$$

The subsolution and supersolution necessary for application of Theorem 5 are equal to *u*_0_ : = *μ*_*ϑ*,1_ and *v*_0_ : = *μ*_*ϑ*,2_, respectively. Since it is easy to see that

(34) $$\begin{array}{c} {T_{\vartheta }\lambda |}_{\left[t_{0},\vartheta -r\right]}={T_{\Theta }\lambda |}_{\left[t_{0},\vartheta -r\right]} \end{array}$$

for Θ ≥ *ϑ* and *λ* ∈ *C*([*t*_0_, Θ], ℝ^*n*^), we have

(35) $$\begin{array}{c} {{\bar{\mu }}_{\vartheta ,j}|}_{\left[t_{0},\vartheta -r\right]}={{\bar{\mu }}_{\Theta ,j}|}_{\left[t_{0},\vartheta -r\right]} \end{array}$$

for Θ ≥ *ϑ*, *j* = 1,2. Thus the functions ${\bar{\lambda }}_{1},{\bar{\lambda }}_{2}\in C([t_{0},\infty ),{\mathbb{R}}^{n})$ defined, for example, by

(36) λ¯j(t):={μ¯ϑ,j(t)for  t∈[t0,ϑ−r],μ¯t+r,j(t)for t∈(ϑ−r,∞),

where *j* = 1,2, *t* ∈ [*t*_0_, *∞*), satisfy

(37) $$\begin{array}{c} \lambda_{1}\left(t\right)\leq {\bar{\lambda }}_{1}\left(t\right)\leq {\bar{\lambda }}_{2}\left(t\right)\leq \lambda_{2}\left(t\right) \end{array}$$

on [*t*_0_, *∞*) and

(38) $$\begin{array}{c} {\bar{\lambda }}_{j}\left(t\right)\equiv {\left(I\left(k,{\bar{\lambda }}_{j}\right)\left(t\right)\right)}^{-1}f\left(t,I{\left(k,{\bar{\lambda }}_{j}\right)}_{t}\right) \end{array}$$

for *j* = 1,2, *t* ∈ [*t*_0_, *∞*). Now choosing, for example, $\lambda :={\bar{\lambda }}_{1}$, and *y* = *I*(*k*, *λ*) (see substitution (21)), the proof is completed since *I* is strictly increasing with respect to the second argument.

**Remark 7**

Tracing the proof of Theorem 6 one can see that assumptions on functional *F* (continuity, quasi-boundedness, and local Lipschitz condition) are not explicitly mentioned. Nevertheless these assumptions can be viewed as almost necessary for validity of inequalities (22) and (23).

## 4. Linear Cases

In this section we apply the nonlinear result given in Section 3 to linear advanced equations. We will prove existence of positive solutions, and we derive asymptotic behavior of positive solutions.

### 4.1. Equation $\dot{y}(t)=(c+d(t))y(t+r)$

Let us consider linear advanced equation (4):

(39) $$\begin{array}{c} \dot{y}\left(t\right)=\left(c+d\left(t\right)\right)y\left(t+r\right), \end{array}$$

where *c* and *r* are positive constants, function *d* : [*t*_0_, *∞*) → ℝ is locally Lipschitz continuous, |*d*(*t*)| < *ɛ* < *c*, and (*c* + *ɛ*) < 1/(*re*).

To apply Theorem 6 we consider two auxiliary scalar linear advanced equations:

(40) x˙(t)=(c−ɛ)x(t+r),z˙(t)=(c+ɛ)z(t+r).

Looking for solutions in the form *x*(*t*) = exp⁡(*λt*) or *z*(*t*) = exp⁡(*λt*) we get corresponding transcendental equations:

(41) $$\begin{array}{c} \lambda =\left(c-ɛ\right)e^{\lambda r}, \end{array}$$

(42) $$\begin{array}{c} \lambda =\left(c+ɛ\right)e^{\lambda r}. \end{array}$$

The following lemma about the real roots of transcendental equations is a simple consequence of Lemma  1 in [5, page  13] (assumption (*c* + *ɛ*) < 1/(*re*) is here substantial).

**Lemma 8**

The following is true:

- equation (41) has just two different positive roots *λ*_*x*_^1^, *λ*_*x*_^2^, *λ*_*x*_^1^ < *λ*_*x*_^2^, and (42) has just two different positive roots *λ*_*z*_^1^, *λ*_*z*_^2^, *λ*_*z*_^1^ < *λ*_*z*_^2^,
- *λ*_*x*_^1^ *r* < 1 and *λ*_*z*_^1^ *r* < 1,
- *λ*_*x*_^2^ *r* > 1 and *λ*_*z*_^2^ *r* > 1,
- *λ*_*x*_^1^ < *λ*_*z*_^1^ and *λ*_*x*_^2^ > *λ*_*z*_^2^.

As a tool for detecting a positive solution of (4), a linear corollary of Theorem 6 is used. We will reformulate the theorem with respect to the case in question; that is, we put

(43) $$\begin{array}{c} n=1,\;\;F\left(t,y_{t}\right):=\left(c+d\left(t\right)\right)y\left(t+r\right) \end{array}$$

(we omit index 1 due to scalar case).

Using substitution (21) we obtain operator (*Tλ*)(*t*) defined by formula (20) in the form

(44) $$\begin{array}{c} \left(T\lambda \right)\left(t\right):=\left(c+d\left(t\right)\right)\exp\left(\int_{t}^{t+r}\lambda \left(s\right)ds\right). \end{array}$$

**Theorem 9**

Let there be continuous functions *λ*_j_* : [*t*_0_, *∞*) → ℝ, *j* = 1,2, *λ*_1_*(*t*) ≤ *λ*_2_*(*t*) for *t* ∈ [*t*_0_, *∞*), satisfying the inequalities

(45) $$\begin{array}{c} \lambda_{1}^{*}\left(t\right)\leq \left(c+d\left(t\right)\right)\exp\left(\int_{t}^{t+r}\lambda_{1}^{*}\left(s\right)ds\right), \end{array}$$

(46) $$\begin{array}{c} \lambda_{2}^{*}\left(t\right)\geq \left(c+d\left(t\right)\right)\exp\left(\int_{t}^{t+r}\lambda_{2}^{*}\left(s\right)ds\right) \end{array}$$

on [*t*_0_, *∞*). Then there exists a solution *y* = *y*(*t*) of (4) on [*t*_0_, *∞*) such that

(47) $$\begin{array}{c} \exp\left(\int_{t_{0}}^{t}\lambda_{1}^{*}\left(s\right)ds\right)\leq y\left(t\right)\leq \exp\left(\int_{t_{0}}^{t}\lambda_{2}^{*}\left(s\right)ds\right). \end{array}$$

**Proof**

Firstly we verify conditions (22) and (23) of Theorem 6. Due to properties of functions *d*, *λ*, inequalities

(48) $$\begin{array}{c} \left|d\left(t\right)\right|<c,\;\left|d\left(t\right)-d\left(t^{\prime }\right)\right|\leq L_{d}\left|t-t^{\prime }\right| \end{array}$$

hold for every *t*, *t*′ ∈ [*t*_0_, *∞*), where *L*_*d*_ is a suitable constant. The boundedness of operator *T* (condition (22)) is on [*t*_0_, *∞*) obvious:

(49) $$\begin{array}{c} \left|\left(T\lambda \right)\left(t\right)\right|=\left|\left(c+d\left(t\right)\right)\exp\left(\int_{t}^{t+r}\lambda \left(s\right)ds\right)\right|<K_{1}:=2ce^{rM}. \end{array}$$

Now, condition (22) holds. Let us verify condition (23). At first we estimate the difference

(50) |∫tt+rλ(s)ds−∫t′t′+rλ(s)ds| =|∫tt+rλ(s)ds+∫t′+rt′λ(s)ds| =|∫t′+rt+rλ(s)ds−∫t′tλ(s)ds| ≤M|t−t′|+M|t−t′|=2M|t−t′|.

Now we have

(51) |(Tλ)(t)−(Tλ)(t′)| =|(c+d(t))exp⁡(∫tt+rλ(s)ds)   −(a+b(t′))exp⁡(∫t′t′+rλ(s)ds)| ≤|d(t)−d(t′)|exp⁡(∫tt+rλ(s)ds)  +d(t′)|exp⁡(∫tt+rλ(s)ds)−exp⁡(∫t′t′+rλ(s)ds)|  +c|exp⁡(∫tt+rλ(s)ds)−exp⁡(∫t′t′+rλ(s)ds)| <[LderM+2ceξ2M]|t−t′|

with the value *ξ* lying between

(52) $$\begin{array}{c} \int_{t}^{t+r}\lambda \left(s\right)ds,\;\;\int_{t^{\prime }}^{t^{\prime }+r}\lambda \left(s\right)ds. \end{array}$$

Since obviously

(53) $$\begin{array}{c} e^{\xi }\leq e^{rM}, \end{array}$$

we get finally

(54) $$\begin{array}{c} \left|\left(T\lambda \right)\left(t\right)-\left(T\lambda \right)\left(t^{\prime }\right)\right|\leq K_{2}\left|t-t^{\prime }\right| \end{array}$$

with

(55) $$\begin{array}{c} K_{2}:=\left(L_{d}+4cM\right)e^{rM}. \end{array}$$

Inequality (23) is verified. Validity of condition (ii) follows from inequalities (45), and (46), and condition (iii) holds in view of the form of the operator *T* (see (44)).

**Theorem 10**

Equation (4) has a positive solution *y* = *y*(*t*) on [*t*_0_, *∞*) satisfying inequalities

(56) $$\begin{array}{c} e^{\lambda_{x}^{1}t}\leq y\left(t\right)\leq e^{\lambda_{z}^{1}t}, \end{array}$$

where *λ*_*x*_^1^, *λ*_*z*_^1^ are defined in Lemma 8.

**Proof**

Without loss of generality we may assume that *t*_0_ is large enough for the asymptotic relations and inequalities to be valid. Now we employ Theorem 9. Let

(57) $$\begin{array}{c} \lambda_{1}^{*}\left(t\right):=\lambda_{x}^{1},\;\lambda_{2}^{*}\left(t\right):=\lambda_{z}^{1}\left(t\right),\;t\in \left[t_{0},\infty \right). \end{array}$$

First we show that *λ*_1_*(*t*), *λ*_2_*(*t*) satisfy inequalities (45), (46). We have to check whether

(58) $$\begin{array}{c} \lambda_{x}^{1}\leq \left(c+d\left(t\right)\right)\exp\left(\int_{t}^{t+r}\lambda_{x}^{1}ds\right). \end{array}$$

Let us simplify and estimate the right-hand side:

(59) $$\begin{array}{c} ℛ=\left(c+d\left(t\right)\right)e^{\lambda_{x}^{1}r}>\left(c-ɛ\right)e^{\lambda_{x}^{1}r}=\lambda_{x}^{1}. \end{array}$$

That is, inequality (45) is fulfilled. Similarly

(60) $$\begin{array}{c} \lambda_{z}^{1}=\left(c+ɛ\right)e^{\lambda_{z}^{1}r}>\left(c+d\left(t\right)\right)\exp\left(\int_{t}^{t+r}\lambda_{z}^{1}ds\right). \end{array}$$

Thus inequality (46) is valid too. Since all the assumptions of Theorem 9 are fulfilled, for solution *y*(*t*) inequalities (56) hold.

### 4.2. Equation $\dot{y}(t)=\gamma (t)y(t+r)$

Let us consider linear advanced equation (5):

(61) $$\begin{array}{c} \dot{y}\left(t\right)=\gamma \left(t\right)y\left(t+r\right), \end{array}$$

where *γ* : [*t*_0_, *∞*) → ℝ^+^ is bounded, locally Lipschitz continuous and *r* is a positive constant. This equation can be viewed in a sense as a generalization of (4) (with *c* = 0 and *d*(*t*) = *γ*(*t*)). In the following criterion of existence of positive solutions of (5) it is important that only one of inequalities similar to inequalities (45) and (46) must be valid, namely, the inequality similar to (46).

**Theorem 11**

For existence of a positive solution *y* = *y*(*t*) of (5) on [*t*_0_, *∞*), the existence of a continuous function *λ* : [*t*_0_, *∞*) → ℝ is necessary and sufficient, satisfying inequality

(62) $$\begin{array}{c} \lambda \left(t\right)\geq \gamma \left(t\right)\exp\left(\int_{t}^{t+r}\lambda \left(s\right)ds\right) \end{array}$$

on [*t*_0_, *∞*). The mentioned positive solution *y* = *y*(*t*) satisfies inequalities

(63) $$\begin{array}{c} 1<y\left(t\right)\leq \exp\left(\int_{t_{0}}^{t}\lambda \left(s\right)ds\right). \end{array}$$

**Proof**

*Sufficiency*. The proof uses Theorem 6 and can be performed similarly as the proof of Theorem 9. Therefore we omit the full version of the proof and underline only main points. Inequalities relevant to inequalities (45), (46) are

(64) $$\begin{array}{c} \lambda_{1}^{*}\left(t\right)\leq \gamma \left(t\right)\exp\left(\int_{t}^{t+r}\lambda_{1}^{*}\left(s\right)ds\right),\\ \lambda_{2}^{*}\left(t\right)\geq \gamma \left(t\right)\exp\left(\int_{t}^{t+r}\lambda_{2}^{*}\left(s\right)ds\right) \end{array}$$

on [*t*_0_, *∞*). We set *λ*_1_*(*t*) ≡ 0 and *λ*_2_*(*t*) ≡ *λ*(*t*). Then inequalities (64) turn into

(65) $$\begin{array}{c} 0\leq \gamma \left(t\right)\exp\left(\int_{t}^{t+r}0\;ds\right), \end{array}$$

(66) $$\begin{array}{c} \lambda \left(t\right)\geq \gamma \left(t\right)\exp\left(\int_{t}^{t+r}\lambda \left(s\right)ds\right). \end{array}$$

The first inequality (65) obviously holds, and the second inequality (66) coincides with inequality (62). Then inequality (63) is a consequence of (47). Since the solution is increasing, inequality on the left-hand side of (63) must be sharp. The solution satisfying (63) is obviously positive.

*Necessity*. If a positive solution *y* = *y*(*t*) of (5) on [*t*_0_, *∞*) exists, we set

(67) $$\begin{array}{c} \lambda_{1}^{*}\left(t\right)\equiv \lambda_{2}^{*}\left(t\right)=\frac{\dot{y}\left(t\right)}{y\left(t\right)}. \end{array}$$

Then both inequalities (64) hold simultaneously since for *i* = 1,2

(68) λi∗(t)=y˙(t)y(t)=γ(t)y(t+r)y(t)=γ(t)exp⁡(∫tt+ry˙(s)y(s)ds)=γ(t)exp⁡(∫tt+rλi∗(s)ds).

Taking *λ*(*t*) in a suitable way we can get sufficient conditions of existence of positive solutions of (5) on [*t*_0_, *∞*). The following corollary illustrates such possibility.

**Corollary 12**

For existence of a positive solution *y* = *y*(*t*) of (5) on [*t*_0_, *∞*) inequality

(69) $$\begin{array}{c} \int_{t}^{t+r}\gamma \left(s\right)ds\leq \frac{1}{e} \end{array}$$

is on [*t*_0_, *∞*) sufficient.

**Proof**

The corollary is a consequence of Theorem 11, where *λ*(*t*) = *eγ*(*t*).

## 5. Concluding Remarks

 Note that criterion (62) of existence of positive solution of advanced equation (5) given by Theorem 11, as a particular case of result for nonlinear system, seems to be independent of the mentioned criterion (9). In recent book [6] there are considered some linear classes of advanced differential equations in Chapter 5. Sufficient conditions of existence of positive solutions are derived. Inequality [6, formula (5.2.2)] contains, in particular, inequality (62) and inequality [6, formula (5.2.3)] contains, in particular, inequality (69).

Results obtained in the paper are sharp in a meaning. Indeed, in accordance with [2,  page 31], [3, page  22] all solutions of (5) oscillate if

(70) $$\begin{array}{c} \underset{t\to \infty }{\lim\;\inf}\int_{t}^{t+r}\gamma \left(t\right)>\frac{1}{e} \end{array}$$

in spite of the fact that inequality (69), guaranteeing the existence of a positive solution, admits the possibility

(71) $$\begin{array}{c} \underset{t\to \infty }{\lim\;\inf}\int_{t}^{t+r}\gamma \left(t\right)=\frac{1}{e}. \end{array}$$

This occurs, for example, for *γ*(*t*) = 1/*er*. It means that inequality (69) in terms of integral average of function *γ* is the best possible.

In connection with the statement of Theorem 10 a problem about the role played by all real roots *λ*_*x*_^1^, *λ*_*x*_^2^, *λ*_*z*_^1^, and *λ*_*z*_^2^ of transcendental equations (41) and (42) arises. Roots *λ*_*x*_^1^, *λ*_*z*_^1^, *λ*_*x*_^1^ < *λ*_*z*_^1^ with properties given in Lemma 8 were used in Theorem 10 to detect a positive solution of (4) satisfying inequalities (56). The role played by the roots *λ*_*x*_^2^, *λ*_*z*_^2^, *λ*_*x*_^2^ > *λ*_*z*_^2^ with properties given in Lemma 8 in the discussion on existence of positive solutions of (4) is not clarified yet. Obviously *λ*_*x*_^2^ and *λ*_*z*_^2^ cannot be used in Theorem 9 to replace *λ*_j_*, *j* = 1,2, in inequalities (45) and (46) (i.e., it is not possible to set *λ*_1_* = *λ*_*z*_^2^, *λ*_2_* = *λ*_*x*_^2^). Nevertheless, as it was demonstrated by advanced equation (6), it has two classes of asymptotically different positive solutions given by inequalities (7). This is the reason why we formulate the following claim.

**Claim 1**

Equation (4) has a positive solution *y* = *y*_2_(*t*) on [*t*_0_, *∞*) satisfying inequalities

(72) $$\begin{array}{c} e^{\lambda_{z}^{2}t}\leq y_{2}\left(t\right)\leq e^{\lambda_{x}^{2}t}, \end{array}$$

where *λ*_*x*_^2^, *λ*_*z*_^2^ are defined in Lemma 8.
